# Characterization of polycrystalline lead oxide for application in direct conversion X-ray detectors

**DOI:** 10.1038/s41598-017-09168-3

**Published:** 2017-08-17

**Authors:** O. Semeniuk, O. Grynko, G. Decrescenzo, G. Juska, K. Wang, A. Reznik

**Affiliations:** 1 Chemistry and materials science program, Lakehead University, 955 Oliver Road, Thunder Bay, ON P7B 5E1 Canada; 2 Advanced detection devices department, Thunder Bay Regional Health Research Institute, 290 Munro Street, Thunder Bay, ON P7A 7T1 Canada; 3 Department of Physics, Lakehead University, 955 Oliver Road, Thunder Bay, ON P7B 5E1 Canada; 4 Department of Solid State Electronics, Vilnius University, Saulėtekio 9 III k., 10222 Vilnius, Lithuania; 5 SYSU-CMU Joint Institute of Engineering, School of Electronics and Information Technology, Sun Yat-sen University, 132 Waihua Rd., Guangzhou, 510006 P. R. China

## Abstract

While polycrystalline lead oxide (poly-PbO) is known to be one of the most promising photoconductors for utilization in X-ray detectors, its major performance parameters such as charge yield and mobility-lifetime product (*μτ*) are still not well established and require further investigation. Combining the conventional X-ray induced photocurrent and pulse height spectroscopy techniques we examine the X-ray photogeneration and recombination processes in poly-PbO. The measurements indicate that the amount of energy required to release a single electron hole pair *W*_±_ (inverse of charge yield) strongly depends on applied electric field and at 10 V/μm reaches ~20 eV/ehp. Fitting the measured pulse height spectra with the Hecht formula provided *μτ* for holes and electrons to be 4.1 × 10^−8^ cm^2^/V and 10^−9^ cm^2^/V, respectively. Obtained *μτ* values combined with recently reported mobility values of charge carriers in PbO suggest a new direction towards improvement of PbO technology by incorporation of Frisch grid or X-ray transistor architectures.

## Introduction

Semiconductor-based direct-conversion X-ray detectors have been actively sought for a wide range of applications in the fields of domestic security, medical imaging and astronomy^1–9^. Compared with its indirect conversion counterpart, the direct conversion scheme offers an improved performance in sensitivity and resolution, provided that a high-performance photoconductor as an X-ray-to-charge transducer is employed. Multiple crystalline and non-crystalline direct-conversion semiconductor materials are currently in competition for practical usage. For large area detectors with a photoconductive layer deposited directly on the imaging array, the crystalline materials are disfavored due to process incompatibility and high thermal budgets^10^. Currently, the only commercially-viable X-ray photoconductor in direct-conversion X-ray imaging is amorphous selenium (a-Se) whose properties meet the requirements of wide dynamic range and low energy applications (due to its comparatively low atomic number Z) and therefore, it is mainly used for medical applications in the so-called “mammography energy range” ~20 keV^11^. For higher X-ray energies, a-Se has to be replaced by higher Z material. With recent results on solution-processed high Z perovskites, it seems like a new generation of smart materials for radiation sensing is emerging, however, large area perovskite semiconductors are not mature enough for practical application and require more investigation^1, 12^. Currently, Polycrystalline lead oxide (PbO) is one of the most promising candidates due to high Z of Pb and proven capability for low-dose and high-resolution imaging. Previously, Simon *et al*. showed the first prototype of a PbO direct conversion flat panel detector and evaluated its imaging performance^13^. The results were very encouraging: the charge yield was high enough for low dose imaging while the modulation transfer function (MTF) was limited only by the pixel size, indicating potentially a very high spatial resolution^13, 14^. However, such a detector exhibited image lag caused by a residual current even after terminating the X-ray irradiation. This is a very undesirable effect that hampers real time imaging applications. The root cause of the residual current is still unclear, although it was suggested that charge accumulation near the bias electrode triggers injection and thus is responsible for the poor temporal performance of the detector^13^. A similar effect was also observed in a-Se structures, where charge trapping on the electrode/a-Se interface was suspected to enhance the local electric field, thus facilitating charge injection^15, 16^. Once X-ray exposure is terminated, the enhancement of electric field decreases as a result of charge de-trapping, which further leads to the decay of the injected current observed as image lag. The injection-related processes in a-Se are relatively slow and were shown to manifest on the order of seconds^15, 16^.

In addition, the X-ray charge yield of PbO, although higher than that of a-Se, was still lower than the theoretically predicted one. This resulted in relatively high electron-hole pair creation energy *W*_±_ The evaluation of *W*_±_ was previously performed with an X-ray induced photocurrent method (XPM)^13, 17^. In this technique, the charge carriers are generated with a relatively *long* X-ray pulse, while constant bias voltage is applied to the detector to extract the generated carriers. *W*_±_ is obtained by integrating the X-ray signal and comparing the total collected charges against the energy of the incident flux of radiation^13, 18^. The major disadvantage of the XPM measurement is that if the charge injection takes place in the material (as it was suggested for poly-PbO), the injected current will be added to the X-ray signal of the detector, thus reducing the calculated *W*_±_ and leading to the overestimated X-ray conversion efficiency of the detector. In addition, the injection can cause the residual current i.e. image lag.

Charge injection is a common problem for many semiconductors^13, 15, 16, 19^ and it was shown that alternative experimental techniques allow an accurate characterization of both the temporal response and the charge yield of the detector. For example, an advanced XPM technique, performed with a sequence of short X-ray pulses (millisecond scale) rather than a single long pulse (seconds scale), is frequently chosen for investigation of the temporal behavior of the detector, since it allows differentiation between injected and X-ray photogenerated charge^15, 16^. At the same time, Pulse Height Spectroscopy (PHS) performed with a monoenergetic X-ray source is also a tool-of choice for accurate measurements of *W*_±_^18, 20–23^. In this technique, the charge from a *single* absorbed X-ray photon is integrated over a very *short* time (microseconds) and compared with the energy of an incident photon. Since the signal is generated by a single photon absorption at very low flux rates (in contrast to X-ray flux used in XPM), there is no significant (if any) electric field redistribution, while utilization of microsecond integration times makes PHS spectrum insensitive to injection currents, thus providing a more accurate measurement of *W*_±_. In addition, the shape of the pulse height spectrum also gives insights into the charge transport, being affected by the mobility-lifetime product, *μτ*, of the carriers^18^. This in turn allows evaluation of the carrier schubweg *s*, i.e., the mean carrier range before it is trapped by deep traps or recombines. Schubweg can be expressed as the product of charge mobility *μ*, lifetime *τ* and applied field *F* i.e. *s* = *μτF*.

It should be noted that PHS measurements have never been performed on poly-PbO mainly due to a challenge to make PbO X-ray detector with low noise to acquire spectra with distinguishable peaks. Recent improvements in the deposition technology lead to a significant dark current suppression that in turn enables measuring an X-ray photocurrent spectrum with distinguishable peaks. In this work, we apply both advanced XPM and PHS methods to study charge generation in polycrystalline PbO to understand the source of lag and to examine the feasibility of real-time imaging. We also provide an analysis of the *μτ* product for holes and electrons (*μ*_*h*_ *τ*_*h*_ and *μ*_*e*_ *τ*_*e*_, respectively) and *W*_±_ for a wide range of electric fields.

## Results

### Experimental data by XPM

**Figure 1:**
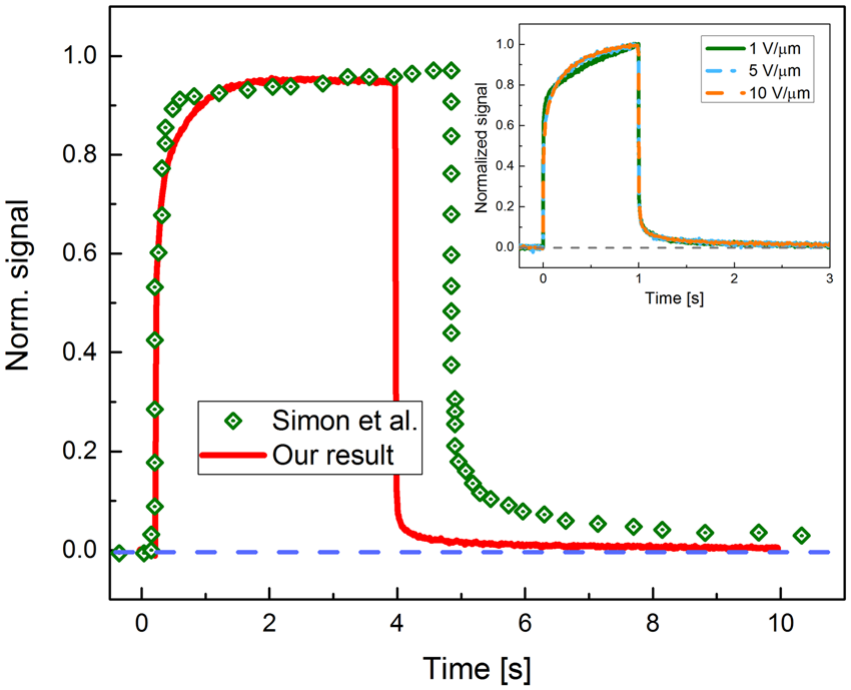
X-ray signal of poly-PbO, measured at *F* = 4 V/μm. The inset shows normalized X-ray signals measured at different exposures. Dark signal *I*_*dark*_ is set to zero.

The typical X-ray response of poly-PbO is shown in Fig. 1. As can be seen, our result is very similar to the previously published data by Simon *et al*.^13^ The amplitude of the signal grows in the beginning of the X-ray pulse, reaching the steady state value in ~2 seconds. At the end of the exposure, there is a well-pronounced residual signal, or lag. With increasing the applied electric field *F*, the signal amplitude and signal lag also increase, however when data are normalized, they closely resemble each other as shown in the inset to Fig. 1.

**Figure 2:**
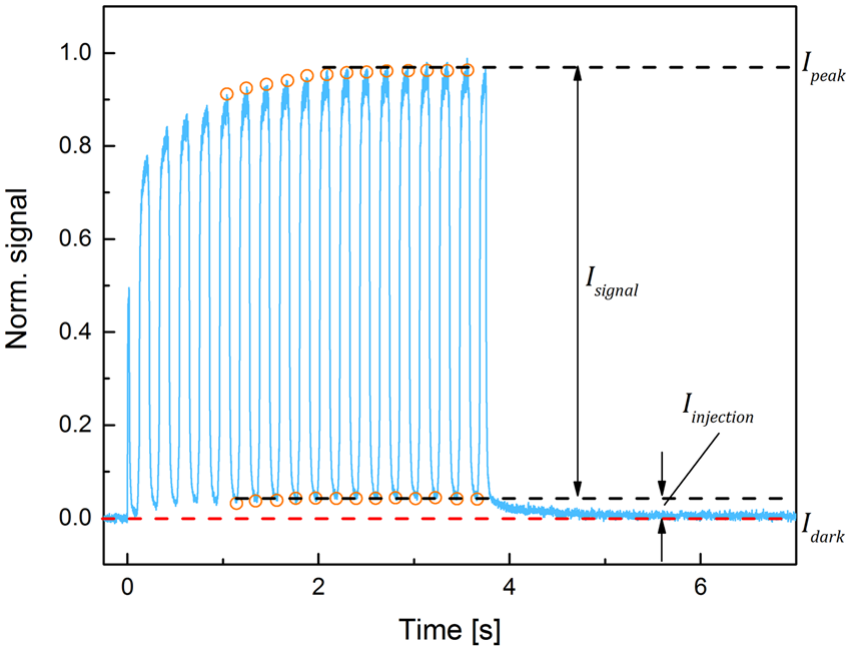
Response of PbO detector to the modulated X-ray exposure at *F* = 2 V/μm. It should be noted that first points at the beginning of the exposure are affected by non-synchronization of the chopper with exposure and fluctuations in X-ray intensity at the beginning of the X-ray pulse.

In order to calculate *W*_±_ from XPM measurements, the signal is integrated and the total collected charged is compared against the exposure to the PbO detector^13, 17^. For the 3.75 s X-ray pulse at *F* = 4 V/μm, we obtained *W*_±_ ≈ 12 eV/ehp, which is larger than theoretically predicted by Klein rule (${W}_{\pm }^{th} \sim \,5.9$ eV)^13^ but it is in a good agreement with the previously reported value of 9.9 eV/ehp, obtained under similar experimental conditions (5 s X-ray pulse, *F* = 3.5 V/μm^13^). However, it should be noted that *W*_±_ measured by this method appeared to be sensitive to the X-ray pulse duration *t*_*pulse*_. Longer X-ray pulses give smaller *W*_±_ and even ${W}_{\pm }^{th}\,$ can be reached at higher electric fields (*F* > 10 V/μm) and longer exposures (*t*_*pulse*_ >  3.75 s). At the same time, if we decrease the X-ray pulse duration, *W*_±_ values will increase. For instance, at *F* = 4 V/μm and *t*_*pulse*_ = 100 ms, *W*_±_ will increase to 14.2 eV/ehp. If we decrease exposure time even further at the same electric field, *W*_±_ values will continue to grow, reaching values of 15.8 and 18.3 eV/ehp at *t*_*pulse*_ = 50 and 10 ms, respectively. In order to investigate such peculiar dependence of *W*_±_ values on the duration of X-ray pulse, we used an X-ray chopper to modulate the exposure to the detector. The example is shown in Fig. 2, where a 3.75 s long X-ray pulse was modulated with the frequency of 5 Hz and 50/50 duty cycle, thus effectively providing ~18 X-ray pulses 100 ms each with the 100 ms interval between pulses.

**Figure 3:**
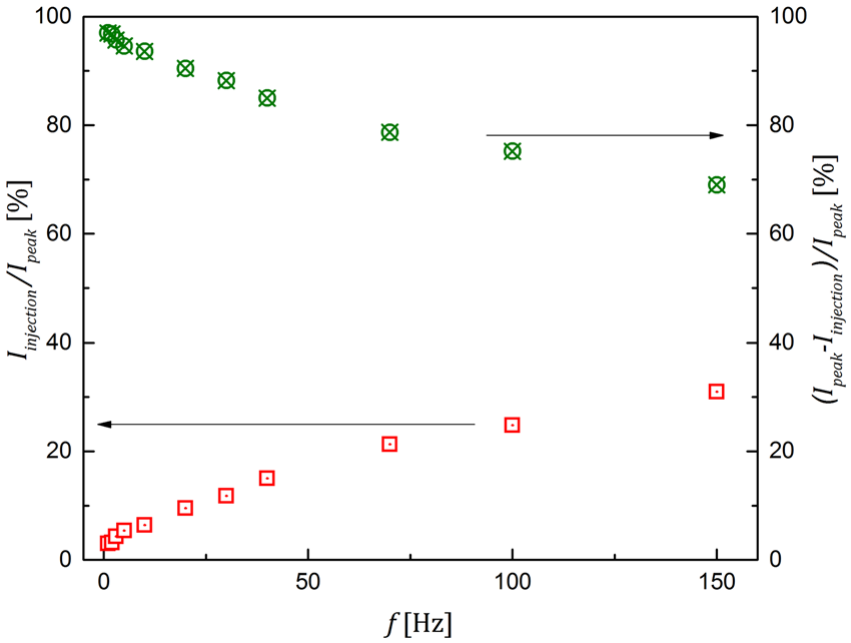
The ratios of *I*_*injection*_/*I*_*peak*_ and *I*_*signal*_/*I*_*peak*_ are plotted as a function of the chopper frequency *f* for *F* = 2 V/μm.

**Figure 4:**
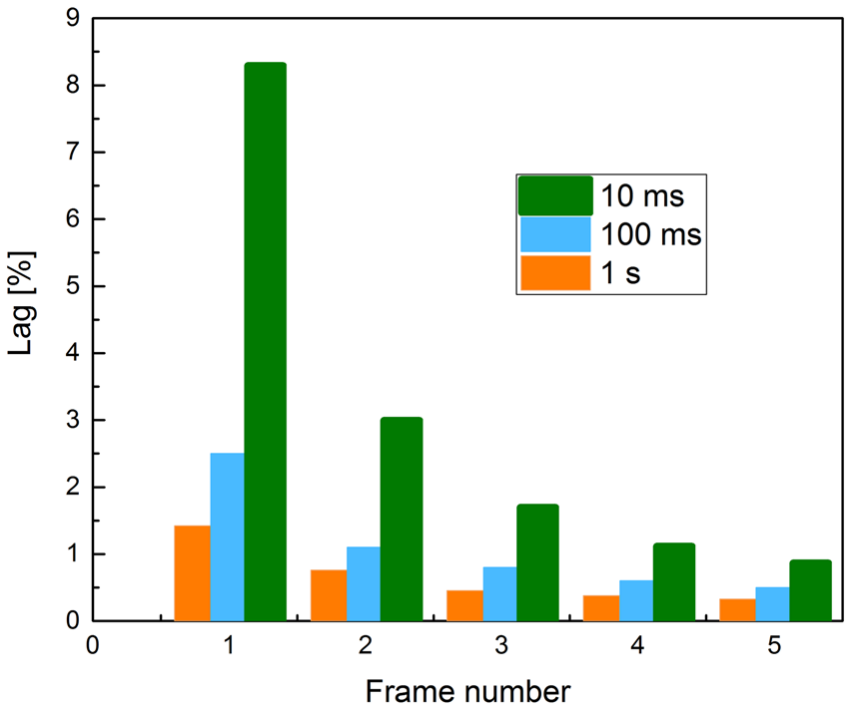
The lag (presented as a percentage of *I*_*peak*_) is shown as the function of a frame number. The frame read-out time is the same as exposure duration.

Experiments with modulated X-ray pulse shown in Fig. 2 revealed that the X-ray response of PbO detector *I*_*peak*_ has two components: the signal due to the X-ray generation of charge carriers *I*_*signal*_ and the signal due to the increase in the dark current i.e. injection *I*_*injection*_. As shown in Fig. 2, *I*_*injection*_ builds up during the X-ray exposure reaching a steady-state value after ~ 2 s, while the *I*_*signal*_ has a constant amplitude. Moreover, it was found that the injection current level changes with the chopper frequency *f* as shown in Fig. 3, where the steady-state ratio of *I*_*injection*_/*I*_*peak*_ is plotted as a function of *f*. At higher frequencies, *I*_*injection*_ contributes more to the total signal (corresponds to higher *I*_*injection*_/*I*_*peak*_ ratio on the graph) than at the lower frequencies. At the end of exposure *I*_*injection*_ decays over time and is observed as lag. As seen from Fig. 2, when X-rays are terminated, lag rolls off from the steady-state *I*_*injection*_ level reached during the X-ray pulses. The decay of lag with time for different frame rates is shown in Fig. 4. The magnitude of lag is proportional to X-ray pulse duration *I*_*pulse*_. Thus, for 1 s X-ray exposure, it takes several seconds to decay below 1%. At the same time if exposure lasts only 10 ms, it takes less than 50 ms to decay to about the same level. Also, lag is more severe at shorter exposures. This is in agreement with the previous observation of higher *I*_*injection*_/*I*_*peak*_ ratio for higher chopper speed (i.e. shorter X-ray pulses) shown in Fig. 3.

### Experimental data by PHS

**Figure 5:**
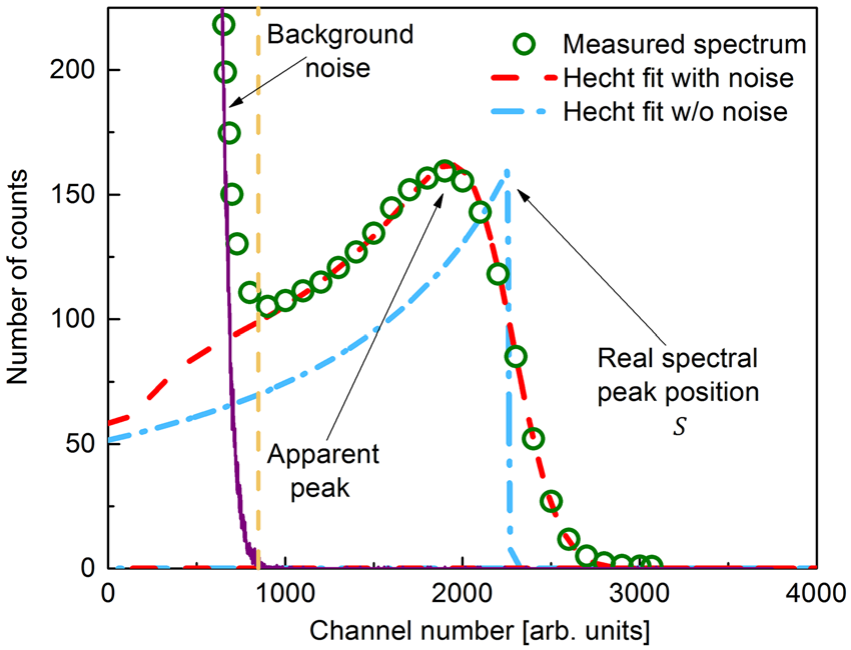
Pulse height spectrum measured with 42 μm PbO sample at *F* = 10 V/μm, *ε* = 59.5 keV, shaping time *τ*_*s*_ = 50 *μ*s and superimposed with noisy and noiseless Hecht fits. Background counts are shown to dominate the spectral waveform below channel 800 (vertical dashed line). The measured position of the noise free spectral peak is shown to be affected by the system noise.

Figure 5 exhibits the typical pulse height spectrum of poly-PbO. The spectral waveform is seen to be asymmetrical and obscured by the background noise at lower channel numbers. The measured PHS allows us to determine the *μτ* product of the charge carriers and the value of *W*_±_ which corresponds to the position of the spectral peak^24^. However, because of asymmetrical nature of the spectrum a numerical simulation must be applied to accurately determine the peak position^24–29^. For our experimental conditions we expect nearly uniform charge generation since the attenuation length of PbO *δ* ≈ 300 μm (for *ε* = 59.5 keV^10^) is much larger than the detector thickness (*d* = 42 μm). Therefore we divided the PbO layers into virtual slices and assuming uniform generation, calculated the collection efficiency from every slice using the depth dependent Hecht formula. This formula describes the collection efficiency *η*(*x*) (i.e., the ratio of collected charge carriers *N*_*col*_ to the total number of carriers created by the X-ray absorption *N*_*gen*_) as follows^24–29^:

1 $$\eta (x)=\frac{{N}_{col}(x)}{{N}_{gen}}={s}_{h}/d(1-{e}^{-\frac{d-x}{{s}_{h}}})+{s}_{e}/d(1-{e}^{-\frac{x}{{s}_{e}}})$$

where *x* is the distance from the anode to the charge generation position, *s*_*h,e*_ is carrier schubweg. Subsequently, the Hecht fit was convolved with noise spectrum and the result was used to fit the measured spectra. After a spectrum fit was obtained the true peak position was determined from the unconvolved Hecht fit (see Fig. 5). The fitting provided the mobility-lifetime products for holes and electrons *μ*_*h*_ *τ*_*h*_ = 4.1 × 10^−8^ cm^2^/V, *μ*_*e*_ *τ*_*e*_ ≈ 10^−9^ cm^2^/V. *μτ* values are assigned to a type of carrier based on the previous reports that revealed holes to be the faster carrier in poly-PbO^30, 31^.

**Figure 6:**
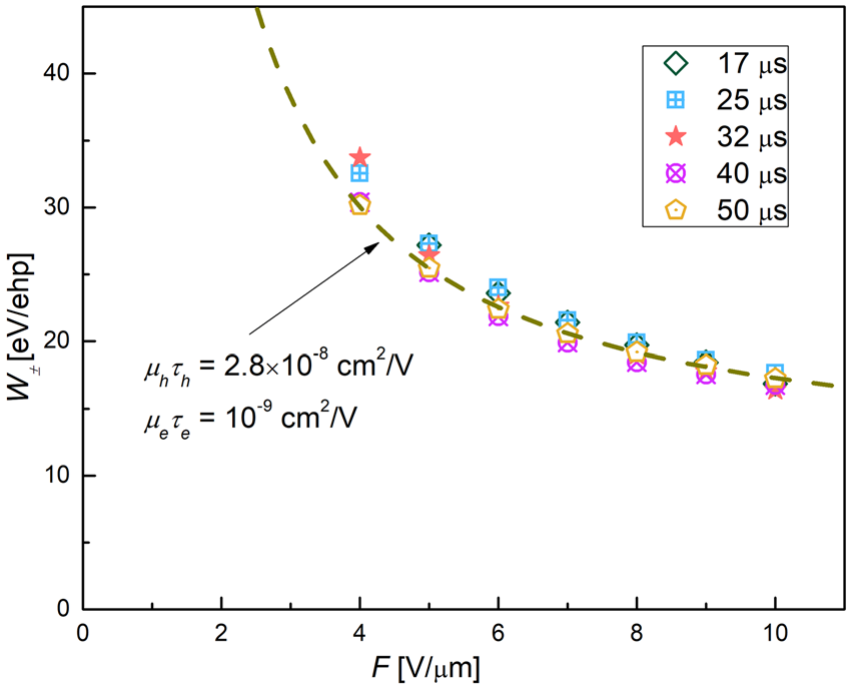
Measured values of *W*_±_ are plotted as a function of *F* for different *τ*_*s*_.

The Hecht fit shows how the true peak position *S* is affected by the system noise, which is typical for materials with significant differences in carrier’s schubweg^24–29^. Electron-hole pair creation energy *W*_±_ was derived from the true peak position *S* as:

2 $${W}_{\pm }=\varepsilon /S,$$

where *ε* = 59.5 keV corresponds to emission from ^241^Am source. Figure 6 shows the field dependence of *W*_±_ for different shaping times *τ*_*s*_, as derived from PHS measurements. As seen from Fig. 6, *W*_±_ decreases with both *F* and *τ*_*s*_ reaching the value ~17 eV/ehp at *F* = 10 V/μm and *τ*_*s*_ = 17 μs. We used the dependence of *W*_±_ on *F* to validate *μτ* values obtained from PHS measurements, following the recipe in ref. 32 where X-ray sensitivity is given by:

3 $$\frac{{N}_{col}}{{N}_{inc}}={x}_{h}[(1-{e}^{-\frac{1}{{\rm{\Delta }}}})+\frac{1}{\frac{{\rm{\Delta }}}{{x}_{h}}-1}({e}^{-\frac{1}{{x}_{h}}}-{e}^{-\frac{1}{{\rm{\Delta }}}})]+{x}_{e}[(1-{e}^{-\frac{1}{{\rm{\Delta }}}})-\frac{1}{\frac{{\rm{\Delta }}}{{x}_{e}}+1}(1-{e}^{-\frac{1}{{x}_{e}}-\frac{1}{{\rm{\Delta }}}})]$$

Here *x*_*e*_, *x*_*h*_ are normalized electron and hole schubwegs, respectively (${x}_{e,h}={s}_{e,h}/d$), Δ is a normalized attenuation length Δ = *δ*/*d* and *N*_*inc*_ is the number of incident X-rays. For our case of uniform absorption, equation (3) can be simplified as follows:

4 $$\frac{{N}_{col}}{{N}_{inc}}=\frac{1}{{\rm{\Delta }}}[{x}_{h}(1+{x}_{h}({e}^{-\frac{1}{{x}_{h}}}-1))+{x}_{e}(1+{x}_{e}({e}^{-\frac{1}{{x}_{e}}}-1))]$$

assuming that for large Δ (i.e. uniform absorption): $(1-{e}^{-\frac{1}{{\rm{\Delta }}}})\to \frac{1}{{\rm{\Delta }}};\,\frac{1}{\frac{{\rm{\Delta }}}{{x}_{e,h}}\pm 1}\to \frac{{x}_{e,h}}{{\rm{\Delta }}};\,{e}^{-\frac{1}{{\rm{\Delta }}}}\to 1$. It should be noted, that equations (3, 4) provide a generic expression of photoconductor sensitivity, simultaneously accounting for both *X-ray absorption efficiency* and *collection efficiency*. To derive the *charge collection efficiency* only as measured with PHS, equation (4) should be divided by absorption efficiency $(1-{e}^{-\frac{1}{{\rm{\Delta }}}})$, which for large Δ reaches $\frac{1}{{\rm{\Delta }}}$. Thus, the expression for collection efficiency for the case of uniform absorption looks as follows:

5 $$\eta =\frac{{N}_{col}}{{N}_{gen}}[{x}_{h}(1+{x}_{h}({e}^{-\frac{1}{{x}_{h}}}-1))+{x}_{e}(1+{x}_{e}({e}^{-\frac{1}{{x}_{e}}}-1))]$$

where ${N}_{gen}={N}_{inc}/{\rm{\Delta }}$. Interestingly, same expression (5) can be obtained by integrating equation (1) over *x* and normalizing by thickness *d*.

Fitting *W*_±_ dependence on *F* (shown in Fig. 6) with equation (5) provided similar *μτ* values as those obtained from spectrum fitting: *μ*_*h*_ *τ*_*h*_ = 2.8 × 10^−8^ cm^2^/V, *μ*_*e*_ *τ*_*e*_ ≈ 10^−9^ cm^2^/V.

## Discussion

Although XPM measurements are affected by the injection current and hence overestimate the total charge created leading to inaccurate *W*_±_ values, they can be still used to elucidate the signal lag in poly-PbO. Conventionally, in disordered structures, lag is linked to the release of carriers that have been previously trapped into deep traps within the band tails^33^. If the release time from these traps is longer than the frame duration, then the de-trapped charge will cause a residual current even after termination of X-ray exposure. However, as seen from the inset of Fig. 1, the lag is scalable with applied field, while Figs. 3 and 4 show that lag is proportional to exposure duration *t*_*pulse*_, indicating that it is not associated with the charge carriers trapping during their transit across the sample. Indeed, if charge trapping and de-trapping was responsible for lag in PbO, the lag should decrease with application of higher electric field, as charge schubweg becomes larger and de-trapping becomes more efficient. The de-trapping time and therefore the signal lag also should not depend on *t*_*pulse*_, as it is a fundamental property of material and not an experimental parameter. Finally, previous observations of lag dependence on the material of the bias electrodes^13^ suggest that charge trapping in the bulk is not the dominant mechanism for signal lag in poly-PbO.

A plausible explanation of these effects is the time-dependent enhancement of the electric field at the metal/semiconductor interface that triggers *injection* from the bias electrodes into the poly-PbO. XPM measurements utilize a flux of X-rays to generate a measurable signal, thus creating a relatively large amount of charge carriers. When the charge carriers get trapped, they redistribute the electric field applied to the sample. If they are trapped on the electrode/photoconductor interface (surface of the photoconductor has an extreme concentration of trapping states), they might cause a local enhancement of electric field. This triggers injection, which builds up over exposure time, and introduces uncertainty in derived values of *W*_±_^15, 16^.

**Figure 7:**
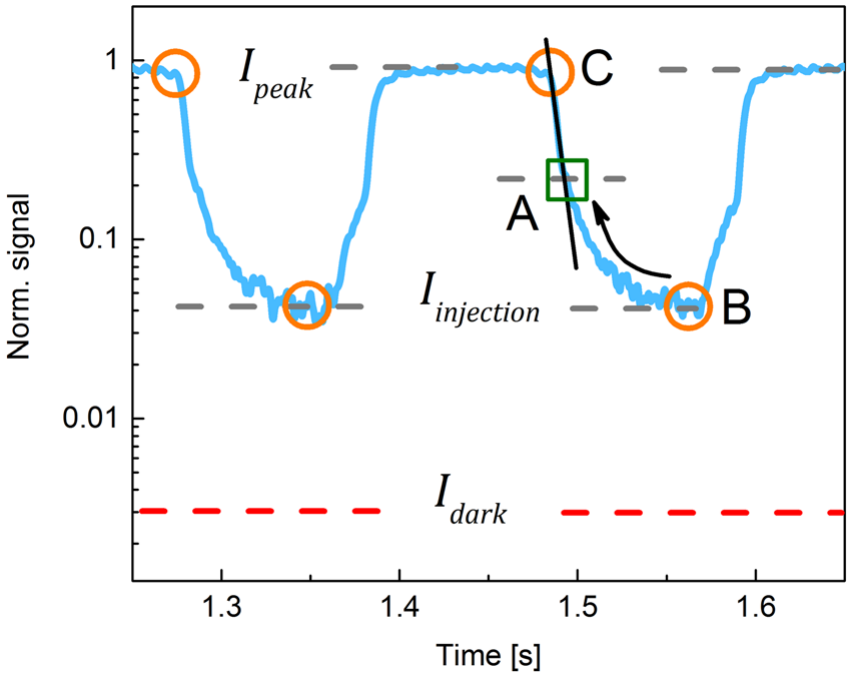
The response of PbO detector to subsequent 100 ms X-ray pulses at *F* = 2 V/μm plotted in the semi-log scale (taken from the middle section of the graph shown in Fig. 2). Points C and B represent the total current and the injection current levels, respectively, while point A defines the inflection point in the signal decay, i.e. the beginning of lag.

The trapped carriers can de-trap and therefore injection builds up until a steady state level of accumulated charge is reached between the trapping and de-trapping charge at the interface. After X-ray exposure, the electric field gradually returns to the initial level and the residual injection current decreases accordingly. The kinetics of the injection build up during exposure is further elaborated with pulsed X-rays. Figure 7 shows the response of poly-PbO to two successive 100 ms pulses, plotted in a semi-log scale.

As shown in Fig. 7, the kinetics of the signal *I*_*signal*_ is composed of two components: fast C-A and slow A-B. The fast C-A component is associated with the transit of the X-ray generated carriers through the sample, while slow A-B component represents the contribution of the injection current to the total signal *I*_*peak*_^15, 16^. With increase in chopper frequency, the X-ray pulse duration and the interval between pulses decrease. This shifts point B toward the A region, thus resulting in larger injection current levels (see Figs. 3 and 4). It would be very tempting to use only the C-A signal value for the *W*_±_ calculations; however, the position of A will vary with experimental conditions such as X-ray pulse duration, exposure, etc. Indeed, as shown in Fig. 4, lag depends on X-ray pulse duration. Since C-A signal value is inversely correlated to lag, it makes C-A also depend on experimental conditions. This suggests that for materials with dynamic type of injection, XPM measurements inherently overestimate *W*_±_ values. Therefore *W*_±_ measurements should be verified with an alternative technique insensitive to injection, such as PHS.

It should be noted, that the XPM measurements performed here indicate that temporal performance of the PbO based detector is limited by the structure of the detector, rather than by fundamental charge transport properties of poly-PbO. This finding suggests a possible avenue of improvement of PbO technology: development a PbO blocking structure, as it was previously done for a-Se^2^. Indeed, sandwiching poly-PbO between the blocking layers that will prevent charge injection from bias electrodes into material, while permitting charge exit from the material, should improve the temporal performance of PbO detectors and make it suitable for real time dynamic imaging.

The asymmetrical shape of the spectral waveform revealed by the PHS measurements and shown in Fig. 5, is indicative of a considerable difference between electron and hole schubwegs and the ballistic deficit as a result of insufficient shaping time^18, 24, 25, 29^. However, an increase in shaping time and the application of higher electric field cannot bring the PHS histogram closer to a Gaussian shape. This suggests that at the electric fields applied here, the transit time of the slower carriers is much longer than the shaping times used. Similar phenomenon was observed on other materials, like CdTe and CdZnTe, which are currently used as gamma-detectors^24, 25, 34^. In these materials, holes are much slower than electrons and possess a relatively long transit time. Also, fitting of the PHS spectra gave a mobility-lifetime products of ≈4.1 × 10^−8^ cm^2^/V (for faster carriers) and ≈10^−9^ cm^2^/V (for slow carrier), respectively. Based on previously reported measurements of electron and hole transport in poly-PbO, we assign the larger *μτ* value for holes. Indeed, it has been shown that similar to a-Se, holes in PbO are much faster carriers than electrons^30, 31^. Given the mobility-lifetime product for holes of *μ*_*h*_ *τ*_*h*_ ≈ 4.1 × 10^−8^ cm^2^/V, at *F* = 10 V/μm the hole schubweg *s*_*h*_ = 41 μm which is almost the sample thickness *d* = 42 μm. It is not surprising that at this particular field charge collection efficiency is no longer dependent on the integration time: as this is seen in Fig. 6 at *F* = 10 V/μm where all data points merge together as most of X-ray generated holes are collected regardless of the integration time used in the experiment. The situation is very much different for slower electrons with *μ*_*e*_ *τ*_*e*_ ≈ 10^−9^ cm^2^/V; they do not contribute significantly to the signal on the μs scale. Overall, obtained with PHS mobility-lifetime values are smaller by an order of magnitude from those reported by Kabir on poly-PbO^13, 35^. The discrepancy may arise from the difference in experimental techniques used to estimate *W*_±_: while in our study we used PHS method, Kabir treated results obtained with XPM technique. Since XPM is sensitive to injection that supplements to the total integrated photocurrent, use of XPM technique may overestimate total collected charge thus underestimating *W*_±_ and overestimating *μτ*.

The *μ*_*h*_ *τ*_*h*_ and *μ*_*e*_ *τ*_*e*_ measured here, allow us to estimate lifetimes for both types of carriers provided mobility values are known. For this purpose, we measured separately the hole mobility using a photo-CELIV technique similar to how it was done in ref. 31 Hole mobility was found to be *μ*_*h*_ ≈ 0.008 cm^2^/Vs at an electric field of 1 V/μm. Measurements at higher electric field were technically unfeasible due to the limited bandwidth of the signal generator. However, for a rough estimate of *τ*_*h*_ we neglect mobility growth at higher electric field, obtaining *τ*_*h*_ = *μ*_*h*_ *τ*_*h*_/*μ*_*h*_ ≈ 5.1 μs. The hole lifetime of *τ*_*h*_ = 5.1 μs differs from the one obtained earlier with the photo-CELIV technique (~ 200 μs)^31^. In photo-CELIV, non-equilibrium carriers are generated in unbiased samples by a short pulse of uniformly absorbed light. After a preset delay time, the carriers are extracted by a linearly increasing voltage pulse. The carrier lifetime is derived by varying the delay time between carrier photogeneration and application of the electric field. Therefore, photo-CELIV provides the lifetime of the *diffusing* carriers that for the special case of poly-PbO can significantly differ from the lifetime of *drifting* carriers. Indeed, the peculiarity of poly-PbO is its spatial inhomogeneity (poly-PbO layers are composed of randomly oriented platelets) that results in hole transport governed by a spatial disorder rather than energy disorder, which is typical for the majority of non-crystalline and amorphous materials^31^. For spatial disorder governed transport, carriers are trapped only when they drift as opposed to the case of energetic disorder where carriers fall into deeper trapping states with time regardless whether they drift or diffuse. Therefore, when holes are not drifting i.e. in the absence of electric field, they diffuse around the generation site and only slowly recombine, since the diffusion length *L* is very small: $L=\sqrt{\mu \tau \frac{kT}{e}}\approx 0.3$ μm (where *k* is Boltzmann constant, *e* – elementary charge and *T* – temperature), in comparison to both the sample thickness and schubweg ~ 41 μm. The relatively high dielectric constant of PbO (*ε* = 13^31^) suggests that the recombination time might be longer than the trapping time during charge transit, thus effectively leading to longer lifetimes measured with CELIV.

In a similar fashion, it is possible to estimate the electron transit time and electron lifetime in our samples. Measurements of electron mobility in such a thick sample required a higher electric field than that could be obtained with the experimental equipment used, therefore as a reference point we used the mobility of electrons reported for a 5 μm thick sample^30^ and extrapolate it to our thickness, as mobility in dispersive media is scalable with the thickness of the material^36, 37^. Since the hole mobility of the 42-μm-thick sample was about an order of magnitude smaller than that of 5-μm-thick sample^31^, we can apply the same ratio to electrons. The electron mobility of the 5-μm-thick sample was found to be *μ*_*e*_ ≈ 2 × 10^−5^ cm^2^/Vs at *F* = 10 V/μm^30^, therefore for 42 μm thick sample we assume electron mobility *μ*_*e*_ ≈ 2 × 10^−6^ cm^2^/Vs. For such *μ*_*e*_ the electron lifetime is *τ*_*e*_ ≈ 0.5 ms, while the electron transit time at maximal *F* = 10 V/μm, would be ~21 ms. These calculations suggest that electrons are responsible for ballistic deficit in the PHS measurements. In order to collect slow electrons i.e. to compensate for a ballistic deficit, the integration time should be on the order of milliseconds. Such a long integration time inevitably leads to significant noise increase^38^ that thus would obscure the signal.

It should be noted that for the shaping times used in the experiment (17–50 μs) the measured *W*_±_ values are seen to saturate at *W*_±_ ≈ 17 eV/ehp for an electric field of 10 V/μm. Such a value of *W*_±_ compares very favorably with a *W*_±_ of ≈45 eV/ehp obtained on a-Se^18, 20^ for the same electric field and shaping times used. a-Se is the only photoconductor currently used in direct conversion mammographic X-ray detectors. Poly-PbO with its higher charge yield and higher quantum efficiency looks to be a promising candidate to replace a-Se in the next generation digital X-ray detectors. In comparison with another promising material, namely HgI_2_, PbO offers a similar performance in terms of signal lag and *W*_±_, while *μτ* and dark current are better in HgI_2_. The later can be improved by developing a PbO blocking structure, as it was successfully done for a-Se^16^. However, HgI_2_ layers are toxic in the sense that they chemically react with imaging array electronics, thus creating additional technological challenges^39, 40^. Currently, the major competitors of PbO seems to be perovskite photoconductors, for example methylammonium lead iodide (CH_3_NH_3_PbI_3_). This photoconductor combines comparatively high carrier mobility with low electron-hole creation energy of ~0.4 eV/ehp caused by photoconductive gain^12^. However, despite high carrier mobility, *μτ* product is still low (although higher than in PbO) due to the short carrier lifetime. In addition, materials with photoconductive gain are generally challenging for real-time imaging, when fast temporal response is needed.

One of the current challenges in poly-PbO is the presence of the slower carrier, which diminishes the temporal response of the material. The co-existence of both fast and slow carriers is a common issue of solid state detectors. It has been addressed by the Frisch grid technology^41^ and X-ray transistor technology^42^. Both technologies take advantage of the motion of the faster carrier locally. The objective is achieved by means of utilizing a coplanar grid, placed close to one of the bias electrodes or using transistor-type architecture. The Frisch grid detector is arranged in such a way that the charge signal is only induced during the motion of the charge carrier between the grid and the collection electrode, thus discarding the transit of the slower carriers across the sample and simultaneously suppressing the effect of charge carriers trapping in the bulk of the sample. The technology has proven efficient for both gas filled^41^ and solid stated^43^ detectors. Therefore, we suggest this proven technological solution be applied to a poly-PbO detector as well. The X-ray transistor architecture on the other hand, utilizes strong lateral field to collect fast carriers, while minimizing the impact of slow carriers on the output photocurrent. We believe that the improved speed of operation of the PbO detector together with smaller values of *W*_±_ and higher quantum efficiency than a-Se will make it a very promising candidate for both radiographic and fluoroscopic X-ray medical imaging applications.

## Methods

### Sample preparation

A 42 μm-thick poly-PbO sample was grown by a thermal evaporation method where a high purity PbO powder was evaporated in the presence of oxygen gas as described elsewhere^13^. Evaporated poly-PbO layers have an interesting structure: it is composed of a porous network of platelets 1–3 μm in size and ~100 nm thick. The samples were deposited on a 25 × 25 mm^2^ glass substrate coated with conductive Indium Tin Oxide (ITO) layer, which serves as anode during XPM and PHS measurements. For the cathode, a gold contact 2 mm in diameter was sputtered atop the PbO layer.

### Experimental apparatus

**Figure 8:**
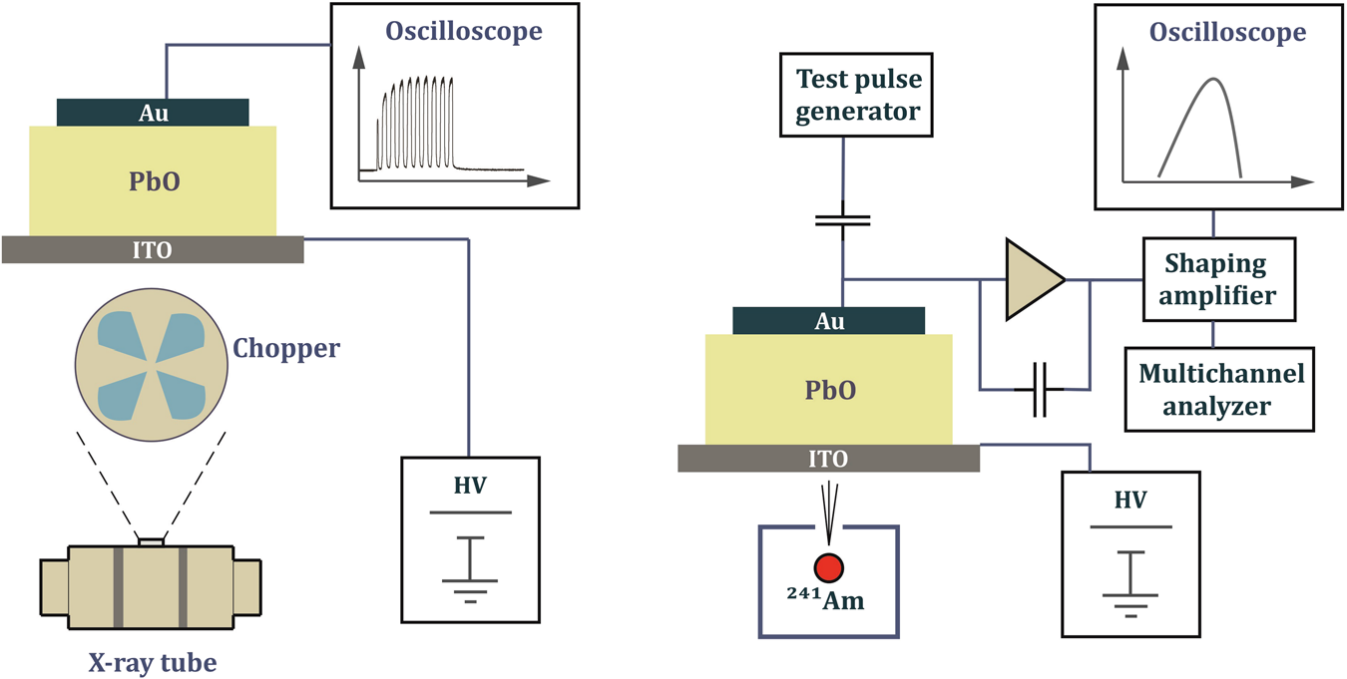
Schematic representation of the XPM apparatus (**a**) and PbO-based pulse height spectrometer with a virtual ground input to charge amplifier (**b**). The exposure was applied from the ITO side.

Both *W*_±_ and *μτ* product in poly-PbO were investigated by X-ray induced photocurrent (XPM) and pulse height spectroscopy (PHS) techniques. The typical XPM and PHS experimental apparatuses are shown in Fig. 8a and b, respectively. For the XPM measurements, the Mammomat 3000 system at 35 kVp was used to generate long (*t = *3.75 s) exposures, while an X-ray tube model PX1412CS at 70 kVp was used to generate less than 1 s exposures. With both systems we utilized a 2 mm thick aluminum filter, which limited the range of energies incident on the detector. The ionization chamber Keithley model 96035 was used to measure X-ray exposure to the poly-PbO detector, which varied between 5 mR to ~1 R, depending on the pulse duration and X-ray system used. Also, a rotating chopper (2 mm Cu) was used to modulate the exposure in a wide frequency range between 1 to 150 Hz. During all experiments, an external power supply maintained a constant bias applied to the sample with the positive polarity applied to ITO. The signal current induced by the X-ray pulse in the PbO layers was observed on a 150 MHz bandwidth digital oscilloscope Tektronix model TDS 420.

For the PHS measurements, an ^241^Am source was used to generate 59.5 keV monoenergetic X-rays. The signal generated by each X-ray photon was readout from the top (Au) contact coupled to a low noise charge preamplifier (AMPTEK model A250CF), then to a pulse shaping amplifier (APTEC model 6300) and finally to a multi-channel analyzer (MCA, Ortec model ASPEC927). At the energy *ε* = 59.5 keV emitted by the ^241^Am source the probability of photoelectric interactions in PbO is 89.1%, meaning that the measured PHS peak represents full absorption of the incident photon energy. The PHS apparatus was calibrated with a Si PIN photodiode (Thorlabs model FDS010). A value of *W*_±_ for Si = 3.62 eV/ehp^18^ was assumed for the photodiode. A HP model 8111 A signal generator, coupled to the test input of the preamplifier was used for the measurement of the preamplifier noise and shot noise intrinsic in the dark current of the detector. The main source of electronic noise was found to be the white noise due to the dark current of the PbO spectrometer, which was found to be *I*_*dark*_ ≈ 200 pA/mm^2^ at 10 V/μm. The main sources of X-ray noise are Compton backscattering due to the glass substrate, aluminum box and the presence of unfiltered low energy peaks in the source itself. The source was collimated to a small beam directly on the sample in order to reduce the stray scatter from adjacent structures. The radioactive source was placed in a lead enclosure shown in Fig. 8b. The impact of the low-energy X-rays from the source was minimized by the use of a 2 mm Al filter placed between the source and the detector. Vibration isolation was used in order to eliminate the influence of external mechanical disturbances. The count rate of ~30–50 s^−1^ ensured that the influence of pulse pile-up can be ignored for pulse shaping time (17–50 μs) used in this study. The *background noise* spectrum was separately measured without the source and used for the deconvolution of the measured spectra.

PHS measurements have been done for the electric fields *F* from 4 V/μm to 10 V/μm with a step of 1 V/μm and for different shaping times *τ*_*s*_ from 17 μs to 50 μs. The lower limit of applied field was determined by the ability to resolve the signal above electronic noise, whereas the upper limit was determined by the increase of the dark current and noise associated with it.
